# College Commencements

**Published:** 1894-04

**Authors:** 


### COLLEGE COMMENCEMENTS.

The exercises were held at the Grand Opera House on the evening of March 6, 1894. Rev. C. P. Williamson made the opening prayer.

Dr. W. S. Kendrick, the Proctor, then read his report, of which the following is a portion :

The session closing had been one of unusual prosperity and success. Amply equipped in all its departments, the college had maintained its high rank and position as worthy the patronage so generously bestowed. Adhering strictly to the rules and rates prescribed in her catalogue, keeping pace with all recent scientific advancement, and with magnificent facilities for both didactic and clinical teaching unparalleled success had attended the session of 1893–1894.

The matriculation book showed an enrollment of three hundred students in the various departments. Of these there were, from Georgia, 199; Alabama, 28; South Carolina, 20; Texas, 12; Florida, 11 , Mississippi, 10; North Carolina, 8; Louisiana, 4; Virginia, 3; Arkansas, 2; Massachusetts, 1; Illinois, 1; Canada, 1. Of this number sixty-six in the medical class and three in the pharmaceutical class were entitled to their diplomas.

The degrees were then conferred by Hon. N. J. Hammond, President of the Board of Trustees.

Dr. Frank M. Ridley, of LaGrange, then delivered the annual address. His speech was appropriate and his delivery showed that his fame as an orator was deserved.

[THE JOURNAL had hoped to publish Dr. Ridley's address in full, but was unable to get it owing to his having spoken only from notes, which we did not anticipate.]

Dr. J. L. Roberts, the valedictorian, delivered an address appropriate to the occasion, and in a pleasing and eloquent manner.

Mr. George Westmoreland presented the prizes as follows:

Dr. Moses G. Campbell, first honor; Dr. William P. Henry, second honor; Dr. Olin H. Weaver, third honor; Drs. J. M. Moore, C. E. Pickett, J. W. Dorough, H. F. Frederick and J. H. Asher received *honorable mention.*

A very large audience witnessed the exercises. The arrangements were elegant and flowers were in profusion.

The commencement exercises of the Southern Medical College were held in DeGive's Opera House Friday night, March 30th. The Dean of the college, Dr. William Perrin Nicolson, in making his report, said that the session just closed was the first after the inauguration of the new system of three full terms of study of six months each. One year ago the faculty determined upon this step with some apprehension, fearing the increase in requirements would lead many students to seek those institutions still adhering to the effete system of two courses of five months each. The result has been more than gratifying. The usual number of students has been in attendance, and of a better *personnel* than ever before, and to-night the college graduates the largest class of its history in spite of a rejection of six in the final examinations. Since last commencement the college has purchased an elaborate equipment in the way of models and apparatus, and these, with others under contract, enhance greatly the teaching facilities of the institution.

The Southern Medical College will maintain its high stand in favor of a better medical education.

The Rev. Robert S. Barrett, of Atlanta, delivered the address to the graduating class. Dr. W. J. Little delivered the valedictory oration. The following honors were awarded: First honor, Dr. C. P. Ward; second honor, Dr. E. L. Brooks; third honor, Dr. J. B. Diamond. The prize offered by Professor Crichton was awarded to Dr. T. J. McArthur. The prize offered by Professor Roy was awarded to Dr. A. B. Evans. There were forty-two members of the graduating class.

On the evening preceding the commencement an elegant banquet was held at the Aragon Hotel. Toasts were delivered by representatives of the Faculty, the graduating class, and invited guests.

